# An MG53-IRS1-interaction disruptor ameliorates insulin resistance

**DOI:** 10.1038/s12276-018-0099-9

**Published:** 2018-06-06

**Authors:** Jun Sub Park, Hyun Lee, Bo Woon Choi, Seonggu Ro, Doyoung Lee, Jeong Eun Na, Jeoung-Ho Hong, Jae-Seon Lee, Bong-Woo Kim, Young-Gyu Ko

**Affiliations:** 1 Division of Life Sciences, Korea University, Seoul, Korea; 2 Tunneling Nanotube Research Center, Korea University, Seoul, Korea; 3 CrystalGenomics, Inc., Seongnam-si, Gyeonggi-do Korea; 4 Department of Molecular Medicine, College of Medicine, INHA University, Incheon, Korea

**Keywords:** High-throughput screening, Drug discovery, Type 2 diabetes

## Abstract

Mitsugumin 53 (MG53) is an E3 ligase that induces insulin receptor substrate-1 (IRS-1) ubiquitination and degradation in skeletal muscle. We previously demonstrated that the pharmaceutical disruption of the MG53-IRS-1 interaction improves insulin sensitivity by abrogating IRS-1 ubiquitination and increasing IRS-1 levels in C2C12 myotubes. Here, we developed a novel MG53-IRS-1 interaction disruptor (MID-00935) that ameliorates insulin resistance in diet-induced obese (DIO) mice. MID-00935 disrupted the molecular interaction of MG53 and IRS-1, abrogated MG53-induced IRS-1 ubiquitination and degradation and improved insulin signaling in C2C12 myotubes. Oral administration of MID-00935 increased insulin-induced IRS-1, Akt, and Erk phosphorylation via increasing IRS-1 levels in the skeletal muscle of DIO mice. In DIO mice, MID-00935 treatment lowered fasting blood glucose levels and improved glucose disposal in glucose and insulin tolerance tests. These results suggest that MID-00935 may be a potential muscle-targeting drug candidate for treating insulin resistance.

## Diabetes: lowering glucose levels by targeting protein interactions

A promising drug candidate for type II diabetes targets a protein interaction involved in insulin resistance. In healthy individuals, the insulin receptor substrate 1 (IRS-1) protein stimulates glucose uptake, particularly in skeletal muscles. In patients with diabetes, the interaction of IRS-1 with another protein, MG53, interferes with insulin signaling and causes insulin resistance, resulting in excess glucose in the blood. Drugs for treating diabetes aim to lower glucose levels, but no existing drugs directly target glucose levels in skeletal muscles. Now, Bong-Woo Kim and Young-Gyu Ko at Korea University in Seoul, South Korea, and co-workers have designed a drug that specifically inhibits the IRS-1-MG53 interaction. Oral administration of the drug showed promise in trials on diet-induced obese mice, increasing IRS-1 levels in skeletal muscles, lowering fasting glucose levels and improving insulin sensitivity.

## Introduction

Type 2 diabetes mellitus (T2DM) is a metabolic disorder caused by insulin resistance in the liver, fat, and muscle. Insulin resistance, which does not respond to normal levels of insulin, leads to excessive glucose production by the liver and impaired glucose uptake by adipose tissue and skeletal muscle, eventually resulting in hyperglycemia, hyperlipidemia, and hyperinsulinemia^1^. The main goal of T2DM treatment is to reduce blood glucose levels to normal levels. Many classes of glucose-lowering drugs, such as biguanides, sulfonylureas, thiazolidinediones, and dipeptidyl peptidase-4 inhibitors, are clinically available, but there are no drugs that act directly on skeletal muscle^1, 2^. Skeletal muscle is the major glucose reservoir and accounts for more than 80% of insulin-induced glucose disposal^3, 4^. Improving the insulin sensitivity of skeletal muscle may be an effective approach for treating T2DM.

MG53, also known as TRIM72, is a member of the tripartite motif-containing protein family, which includes three distinct domains: a RING finger, B-Box, and coiled-coil domain^5^. MG53, which is highly expressed in cardiac and skeletal muscle, is transcribed by MyoD and MEF2 during skeletal myogenesis^6^. With the aid of the E2 ubiquitin-conjugating enzyme UBE2H, the MG53 protein acts as an E3 ubiquitin ligase due to its RING domain^7, 8^. Because IRS-1 is a common modulator of both IGF and the insulin signaling pathway, MG53-induced IRS-1 ubiquitination and degradation negatively regulate IGF-induced myogenesis and insulin signaling in skeletal muscle^5, 7^. The genetic disruption of MG53 increases IRS-1 levels, improves insulin signaling in mouse skeletal muscle and ameliorates insulin resistance in diet-induced obese (DIO) mice^7, 9^. Thus, MG53 is a potential therapeutic target for treating insulin resistance.

In addition to the functions of MG53 in insulin signaling, MG53 plays a crucial role in the muscle membrane repair process. During muscle contraction, the sarcolemma is exposed to acute membrane damage. In response to oxidized flux, MG53 promotes vesicle trafficking to damaged sites via interaction with dysferlin and reseals the shredded membrane by oligomerization^10, 11^. MG53-disrupted mice exhibit defective membrane repair in mechanically or chemically induced injuries. Adeno-associated virus-mediated MG53 gene delivery ameliorates muscular dystrophy and heart failure by enhancing membrane repair in δ-sarcoglycan-disrupted hamsters^12^. In addition, the exogenous injection of recombinant MG53 protein protects the muscle, heart, lung, brain, liver, and kidney from membrane damage^13–19^.

Because MG53 plays a positive role in membrane repair but a negative role in insulin signaling, the development of MG53-targeting drugs requires precise mechanism-based manipulation. Specific inhibition of the MG53-IRS-1 interaction can be used as a therapeutic approach for treating insulin resistance without affecting the membrane repair function of MG53^20^. Using a bimolecular luminescence complementation (BiLC) system, we found a small chemical called MG53-IRS-1 interaction disruptor-1 (MID-1). MID-1 interferes with the binding of MG53 to IRS-1, increases IRS-1 levels, and improves insulin signaling in C2C12 myotubes^21^. However, the intraperitoneal injection or oral administration of MID-1 is not effective at improving insulin sensitivity in a DIO mouse model. Various MID derivatives have been screened using BiLC systems to develop effective in vivo MID candidates. Here, we demonstrated that MID-00935 improved insulin signaling by disrupting the binding of MG53 to IRS-1 and abrogating MG53-induced IRS-1 ubiquitination in C2C12 myotubes. In addition, oral administration of MID-00935 improved insulin signaling in skeletal muscle and ameliorated insulin resistance in DIO mice. Based on these findings, we suggest that MID-00935 may be a potential drug candidate for treating insulin resistance via targeting skeletal muscle.

## Materials and methods

### Screening of MID chemicals using the BiLC system

BiLC constructs and a stable cell line for screening MID chemicals were developed as described previously^21^. For the lysate-based BiLC assay, stable cell lysates were incubated with MID chemicals for 1 h at the indicated concentrations. For the cell-based BiLC assay, stable cells were cultured in 6-well plates, incubated with MID chemicals for 24 h and then lysed. Luminescence was measured with a Steady-Glow Luciferase assay kit (Promega, WI, USA) according to the manufacturer’s instructions and a SpectraMAX i3x multiplate reader (Molecular Devices, CA, USA).

### Cell culture and transfection

The HEK 293 cell line and C2C12 myoblasts were maintained as previously described^22^. Briefly, HEK 293 cells and C2C12 myoblasts were grown in DMEM supplemented with 1% penicillin/streptomycin and 10% fetal bovine serum in a 5% CO_2_ incubator at 37°C. C2C12 myotubes were prepared using low passage (5–7) myoblasts, as previously described^22^. HA-MG53, HA-C14A, and Flag-IRS-1 plasmids were generated as previously described^7^. Plasmids were transfected into HEK 293 cells using Lipofectamine 3000 (Invitrogen, CA, USA) according to the manufacturer’s instruction.

### Immunoblotting and co-immunoprecipitation

Immunoblotting and co-immunoprecipitation analyses were performed as previously described^21^. Briefly, immunoblotting samples were prepared with RIPA lysis buffer (50 mM Tris-HCl, 150 mM NaCl, 1% Nonidet P-40, 0.1% SDS, 0.5% sodium deoxycholate and protease and phosphatase inhibitors at pH 7.5). Co-immunoprecipitation samples were prepared with IP lysis buffer (20 mM Tris-HCl (pH 7.5), 150 mM NaCl, 1% Nonidet P-40, 5% glycerol, 1 mM EDTA with protease and phosphatase inhibitors). Then, 500 μg of protein was incubated with each antibody for 24 h at 4°C, followed by incubation with protein A-sepharose or protein G-agarose beads (Roche Applied Science) for 2 h. Samples were subjected to SDS-PAGE and transferred to nitrocellulose membranes. Antibodies against phospho-IRS-1(S302), phospho-IRS-1(S612), Akt, ERK, phospho-Akt, and phospho-ERK were purchased from Cell Signaling Technology, and antibodies against Flag, HA, ubiquitin, GAPDH, and phospho-IRS-1(Y628) were purchased from Santa Cruz Biotechnology (CA, USA). The IRS-1 antibody was purchased from BD Transduction Laboratories (CA, USA), and the phospho-IRS-1(Y608) antibody was purchased from Merck Millipore (MA, USA).

### Quantitative real-time PCR

Total RNA was extracted from C2C12 myotubes or mouse soleus muscle with an RNeasy mini kit (Qiagen, Hilden, Germany). cDNA was synthesized using RT master premix (ELPIS Biotech, Daejeon, South Korea). Gene expression was determined using a LightCycler 480 II instrument with TOPreal qPCR 2× PreMIX (Enzynomics, Daejeon, South Korea) as previously described^7^. Expression levels were normalized to the 18 S or GAPDH expression level and presented as the fold change.

### Cycloheximide chase assay

Four-day differentiated C2C12 myotubes were incubated with 5 μM MID-00935 or 0.1% DMSO for 24 h. Then, the cells were treated with cycloheximide (10 μg/ml, Sigma, MA, USA) and harvested at the indicated time. IRS-1 and MG53 protein levels were determined by immunoblotting.

### Glucose uptake assay

Glucose uptake was measured using a glucose uptake fluorometric assay kit (Biovision, CA, USA). Four-day differentiated C2C12 myotubes were treated with vehicle (0.1% DMSO) or 5 μM MID-00935 for 24 h. Glucose uptake was measured according to the manufacturer’s instructions, as previously described^23^. Insulin-induced glucose uptake was calculated by subtracting the amount of basal glucose uptake from the amount of glucose uptake after insulin treatment.

### Animal experiments

To generate DIO mice, four-week-old C57BL/6 N WT male mice were purchased (Orient Bio, Seongnam, South Korea) and fed a 60% high-fat diet (D12492, Research Diets, NJ, USA) for 10 weeks. After 10 weeks, DIO mice were divided into two groups, and vehicle (10% poloxamer 407 solution containing 30% propylene glycol and 5% soybean oil) or MID-00935 (100 mg per kg body weight, mpk) was orally administered. Oral administration was conducted twice a day, at 9 AM and 9 PM, for 1 to 2 weeks. All experiments were approved by the Korea University Institutional Animal Care & Use Committee (KUIACUC-2017-22).

### Glucose tolerance and insulin tolerance tests

For glucose tolerance tests, overnight-fasted male DIO mice were injected intraperitoneally with d-glucose (2 g/kg of body weight). For the insulin tolerance test, 4-h fasted male DIO mice were injected intraperitoneally with insulin (1.5 U/kg body weight). Blood samples were collected by tail cutting at each time point, and glucose levels were determined with an Accu-Chek Active glucometer (Roche, Basel, Swiss).

### Insulin signaling in the skeletal muscle

To evaluate insulin signaling in the skeletal muscle, insulin (10 U/kg of body weight) was intraperitoneally injected into overnight-fasted DIO mice. After 10 min, soleus muscles were isolated and immediately frozen in liquid nitrogen. Samples were homogenized in ice-cold lysis buffer with a T10 homogenizer (IKA, Staufen, Germany) and were subjected to immunoblotting.

### Measurement of insulin and leptin

Blood samples were collected from overnight-fasted DIO mice and then incubated at room temperature for 30 min. After centrifugation at 1000×*g* for 10 min, the supernatants of the blood samples were separated. Serum insulin and leptin levels were determined with mouse ELISA kits (Millipore, MA, USA).

### Measurement of dietary intake, locomotor activity, energy expenditure, and core body temperature

To determine food uptake and energy expenditure, male DIO mice were housed individually in an Oxylet indirect calorimetry system (Panlab, Barcelona, Spain) at the end of 1 week of vehicle or MID-00935 administration. Then, O_2_ consumption (*V*_O2_) and CO_2_ production (*V*_CO2_) were measured at 3 min intervals for 2 days (1 day of measurement after 1 day of acclimation). Energy expenditure was calculated automatically. Food uptake and locomotor activity were measured according to the manufacturer’s instructions. Core body temperature was measured by a portable digital thermometer with a rectal probe (TK-610B, Harvard apparatus, MA, USA).

### Statistical analysis

All data are presented as the means ± SEM. Statistical analyses were performed using a two tailed Student’s *t*-tests.

## Results

### Screening effective MID derivatives

**Fig. 1:**
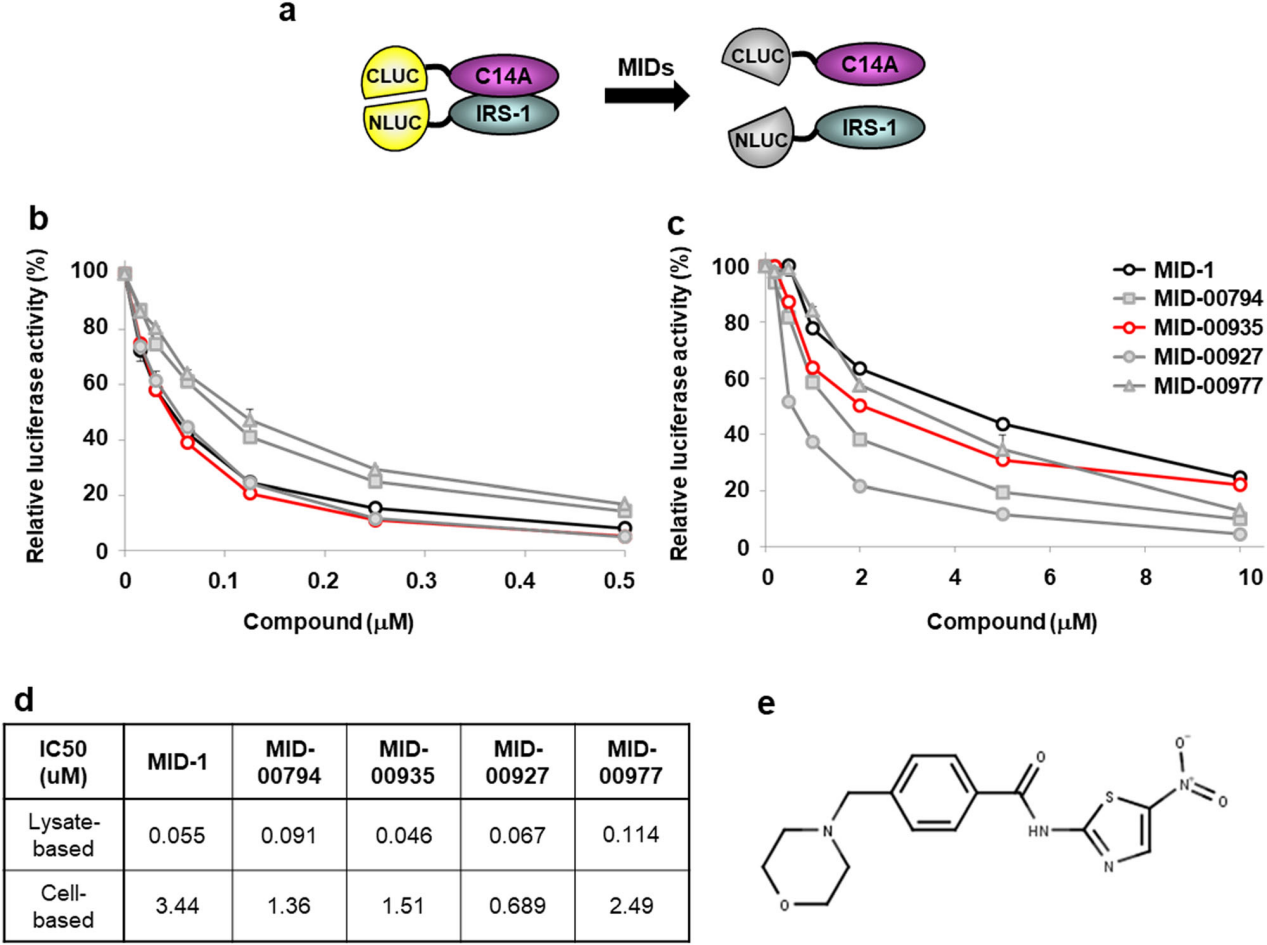
Screening effective MID derivatives. Scheme of the bimolecular luminescence complementation (BiLC) system for screening MID derivatives. Briefly, we used HEK 293 cells expressing a C-terminal luciferase fragment fused with MG53 C14A (CLUC-C14A) and an N-terminal luciferase fragment fused with IRS-1 (NLUC-IRS-1). MID chemical candidates were identified by reduced luciferase activity (**a**). Luciferase activity was measured in lysate- and cell-based BiLC systems after treatment with four MID-1 derivatives (**b**, **c**). The IC_50_ values for luciferase activity were measured in lysate- and cell-based BiLC systems (**d**). Chemical structure of MID-00935; 4-(morpholinomethyl)-N-(5-nitrothiazol-2-yl) benzamide (**e**). Data represent the means ± SEM, *n* = 3 per group in **b** and **c**

We previously established a BiLC system for screening MID chemicals;^21^ this system uses a HEK 293 stable cell line expressing N-terminal luciferase fused with IRS-1 (NLUC-IRS-1) and C-terminal luciferase fused with MG53 C14A (CLUC-C14A) (Fig. 1a). We synthesized ~300 MID derivatives based on the structure of MID-1 [N-(1,3-Thiazol-2-yl) benzamide] and found four MID derivatives that reduced luciferase activity better than MID-1 in the BiLC system. These 4 chemicals exhibited lower IC_50_ values than MID-1 in both lysate- and cell-based assays (Fig. 1b–d). MID-00935 had no cellular toxicity at 10 μM but had in vivo toxicity at 200 milligrams per kg of body weight (mpk); MID-00794 and MID-00977 exhibited cellular toxicity at 10 μM, and MID-00927 had in vivo toxicity at 50 mpk. Thus, we selected MID-00935 [4-(morpholinomethyl)-N-(5-nitrothiazol-2-yl)-benzamide] for further studies (Fig. 1e).

### MID-00935 interferes with the interaction between MG53 and IRS-1 and with MG53-induced IRS-1 ubiquitination

**Fig. 2:**
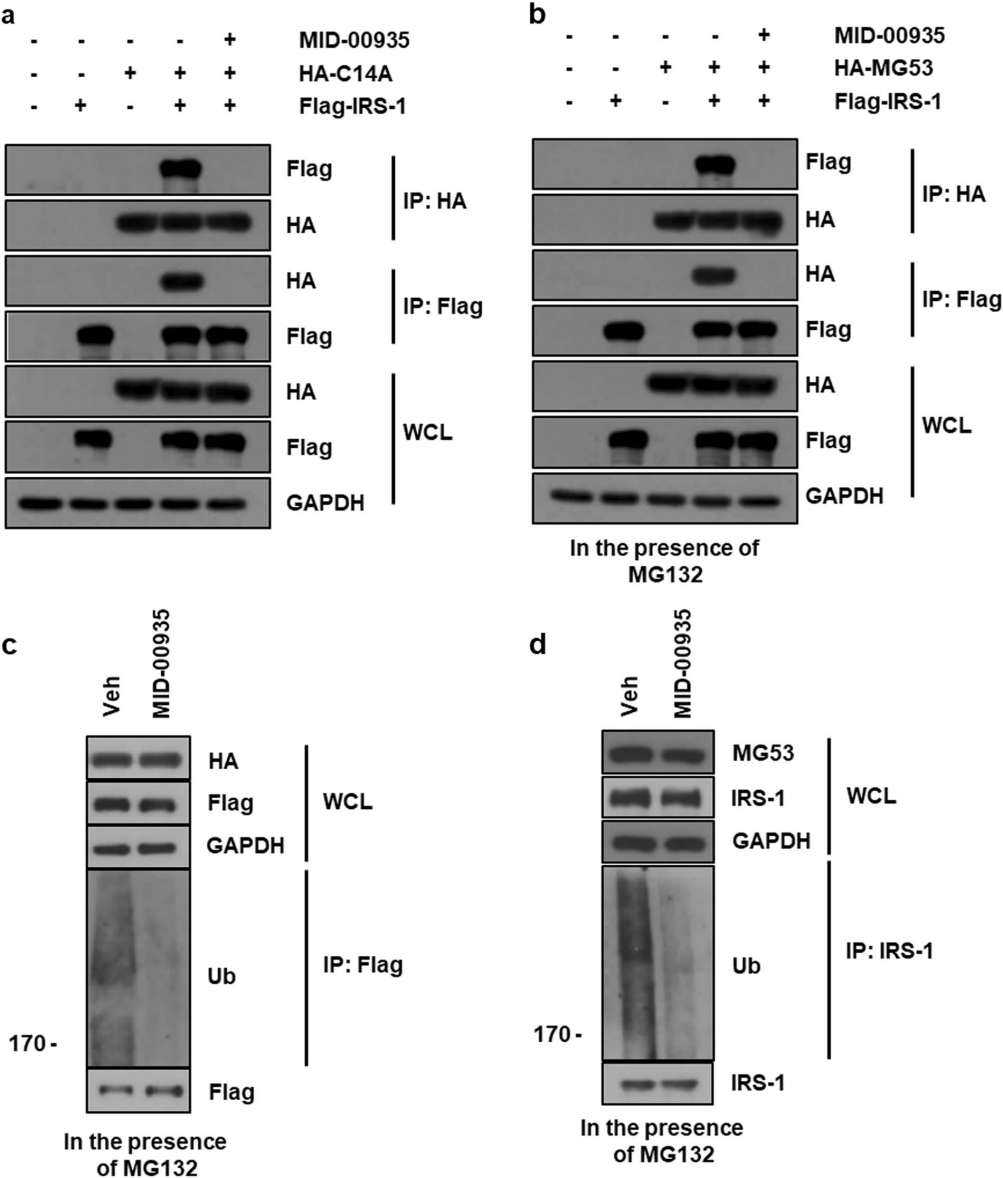
MID-00935 interferes with the molecular interaction between MG53 and IRS-1. HEK 293 cells were transiently transfected with HA-MG53 C14A (C14A, 2.5 μg) and Flag-IRS-1 (2.5 μg) for 24 h and treated with or without MID-00935 (10 μM) for 12 h. The molecular interaction between MG53 and IRS-1 was determined by reciprocal immunoprecipitation (**a**). HEK 293 cells were transiently transfected with HA-MG53 (2.5 μg) and Flag-IRS-1 (2.5 μg) for 24 h and treated with or without MID-00935 (10 μM) in the presence of MG132 (2.5 μM) for 12 h. The molecular interaction between MG53 and IRS-1 was investigated by reciprocal immunoprecipitation (**b**), and IRS-1 ubiquitination was evaluated by ubiquitin immunoblotting after Flag immunoprecipitation (**c**). Four-day differentiated C2C12 myotubes were treated with or without MID-00935 (10 μM) in the presence of MG132 (2.5 μM) for 12 h. IRS-1 ubiquitination was determined by ubiquitin immunoblotting after IRS-1 immunoprecipitation (**d**)

To determine whether MID-00935 interferes with the interaction between MG53 and IRS-1, we performed reciprocal exogenous co-immunoprecipitation assays in HEK 293 cells that transiently overexpressed HA-C14A and Flag-IRS-1 in the presence of MID-00935. It should be noted that because C14A is an inactive E3 ligase mutant, C14A interacts with IRS-1 without inducing IRS-1 ubiquitination and degradation. As shown in Fig. 2a, the molecular interaction between C14A and IRS-1 was absent in the presence of MID-00935. Next, the effect of MID-00935 on the molecular interaction between MG53 and IRS-1 was investigated in HEK 293 cells overexpressing wild-type HA-MG53 in the presence of MG132. As shown in Fig. 2b, reciprocal exogenous immunoprecipitation showed that MID-00935 dissociated IRS-1 from MG53. Next, we determined the effect of MID-00935 on MG53-induced IRS-1 ubiquitination in MG53- and IRS-1-expressing HEK 293 cells and C2C12 myotubes. As shown in Fig. 2c, d, MID-00935 abolished MG53-induced IRS-1 ubiquitination in both exogenous and endogenous systems.

### MID-00935 improves insulin signaling by increasing the IRS-1 level

**Fig. 3:**
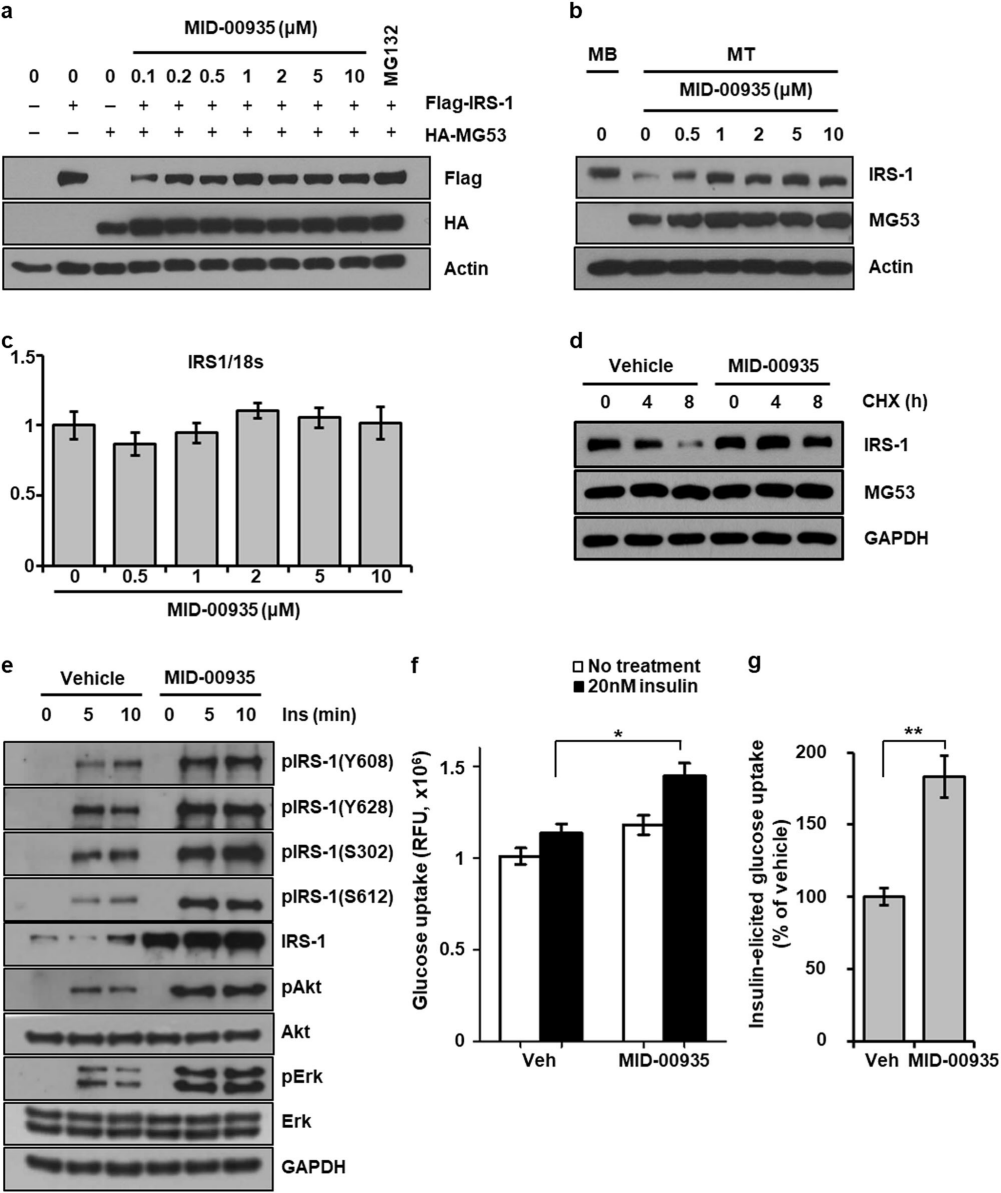
MID-00935 improves insulin signaling by increasing IRS-1 levels. HA-MG53 and Flag-IRS-1 were transiently transfected in HEK 293 cells for 24 h, and cells were treated with the indicated concentrations of MID-00935 or MG132 (2.5 μM) for 12 h. Flag-IRS-1 and HA-MG53 levels were determined by immunoblotting (**a**). Four-day differentiated C2C12 myotubes were treated with the indicated concentrations of MID-00935 or vehicle (0.1% DMSO) for 24 h. IRS-1 and MG53 protein levels were determined by immunoblotting (**b**). The mRNA level of IRS-1 was determined by qPCR. (*n* = 3) (**c**). Four-day differentiated C2C12 myotubes were pre-incubated with 0.1% DMSO or 5 μM MID-00935 for 24 h and then treated with cycloheximide (CHX) for 0, 4, or 8 h. The protein levels of IRS-1 and MG53 were determined by immunoblotting (**d**). Four-day differentiated C2C12 myotubes were treated with MID-00935 (5 μM) for 24 h and stimulated with 20 nM insulin for 0, 5, and 10 min. The protein levels of MG53, IRS-1, p-IRS-1(Y608, Y628, S302, S612), Akt, p-Akt, Erk1/2, p-Erk1/2, and GAPDH were determined by immunoblotting (**e**). Four-day differentiated C2C12 myotubes were incubated with vehicle (0.1% DMSO) or 5 μM MID-00935 for 24 h, stimulated with 20 nM insulin for 20 min and then incubated with 2-DG (10 mM) for 20 min. Glucose uptake is presented as cell-associated fluorescence (**f**), and insulin-induced glucose uptake is presented as a percentage of that in the vehicle-treated group (**g**). Data represent the means ± SEM; *n* = 3; *p < 0.05, **p < 0.01

To examine the effect of MID-00935 on IRS-1 expression levels, IRS-1 levels were measured in HEK 293 cells expressing MG53 and IRS-1 after treatment with different concentrations of MID-00935. Figure 3a shows that treatment with MID-00935 progressively increased IRS-1 levels in a concentration-dependent manner. The effect of MID-00935 on the endogenous IRS-1 level was also examined in C2C12 myotubes. As shown in Fig. 3b, IRS-1 levels increased proportionally with the MID-00935 concentration. In both HEK 293 and C2C12 myotubes, 1 μM MID-00935 restored IRS-1 levels in MG53-expressing cells to the levels in vehicle-treated cells. In addition, MID-00935 did not affect the mRNA level of IRS-1 (Fig. 3c) and increased the half-life time of IRS-1 in C2C12 myotubes in the presence of cycloheximide (Fig. 3d). These results suggest that MID-00935 prevents MG53-induced IRS-1 degradation rather than IRS-1 transcriptional upregulation. Because MID-00935 restored IRS-1 levels in MG53-expressing cells, MID-00935 might improve insulin signaling. To address this hypothesis, we measured the insulin-induced phosphorylation levels of IRS-1, Akt, and Erk after treating C2C12 myotubes MID-00935. MID-00935 increased the insulin-induced phosphorylation levels of IRS-1, Akt, and Erk and increased the IRS-1 level (Fig. 3e). We also examined the effect of MID-00935 on insulin-induced glucose uptake in C2C12 myotubes. As shown in Fig. 3f, g, MID-00935 increased insulin-induced glucose uptake by 1.8-fold. These data indicate that MID-00935 improves insulin sensitivity by increasing IRS-1 levels in C2C12 myotubes.

### MID-00935 ameliorates insulin resistance in DIO mice

**Fig. 4:**
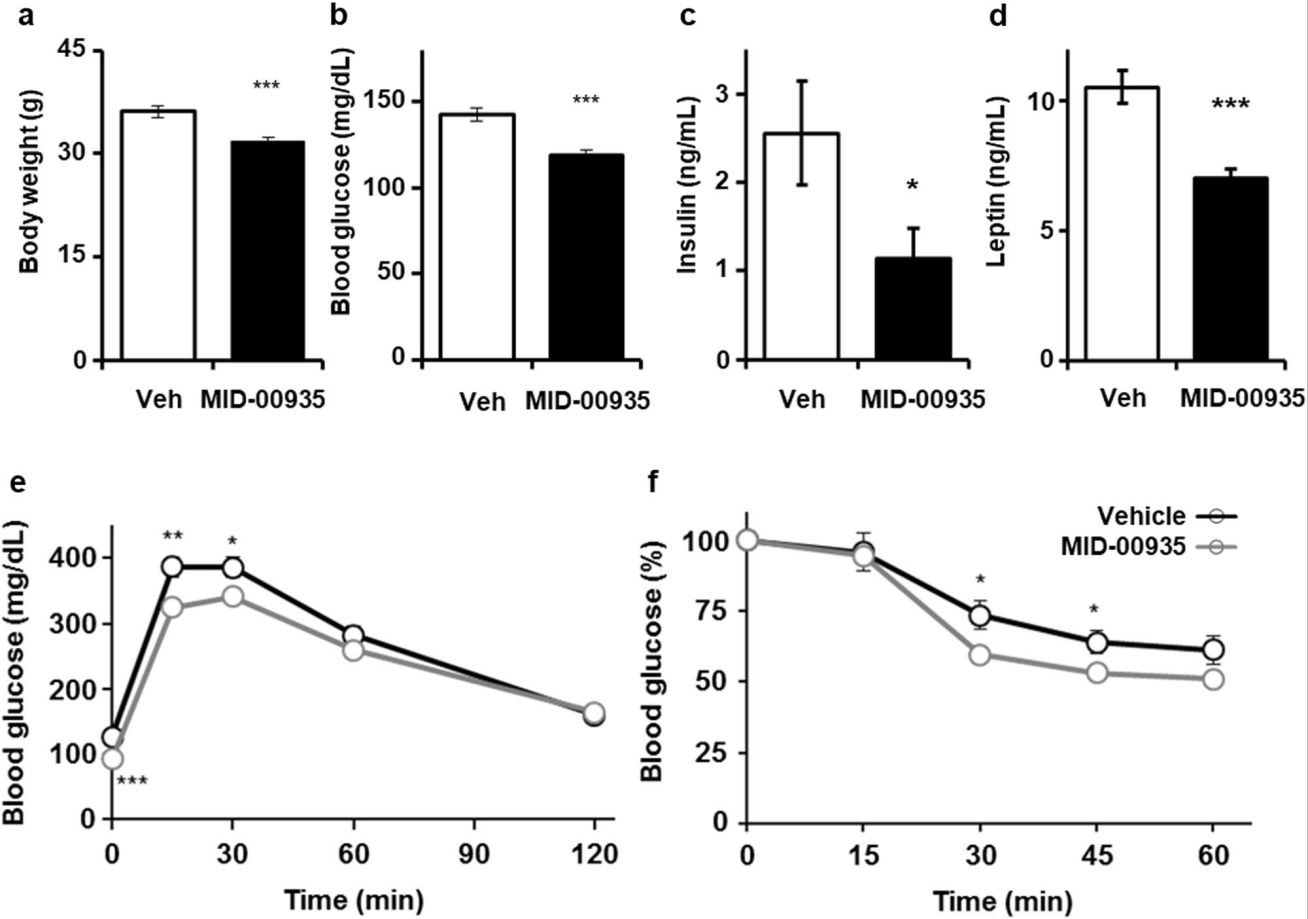
MID-00935 treatment ameliorates insulin resistance in DIO mice. Four-week-old male mice were fed a high-fat diet for 8 weeks and orally treated with vehicle or MID-00935 (100 mpk, twice a day) for one week. Body weights and fasting blood glucose levels were measured (**a**, **b**). Serum was obtained from overnight-fasted mice. Serum insulin and leptin concentrations were determined (*n* = 12 for each group) (**c**, **d**). Four-week-old male mice were fed a high-fat diet for 10 weeks and orally treated with vehicle or MID-00935 (100 mpk, twice a day) for one week. Glucose tolerance tests were performed in DIO mice after 1 week of MID-00935 treatment, and insulin tolerance tests were performed in DIO mice after 2 weeks of MID-00935 treatment (*n* = 10 for each group) (**e**, **f**). Data are presented as the means ± SEM. *t*-test; *p < 0.05, **p < 0.01, ***p < 0.001

**Fig. 5:**
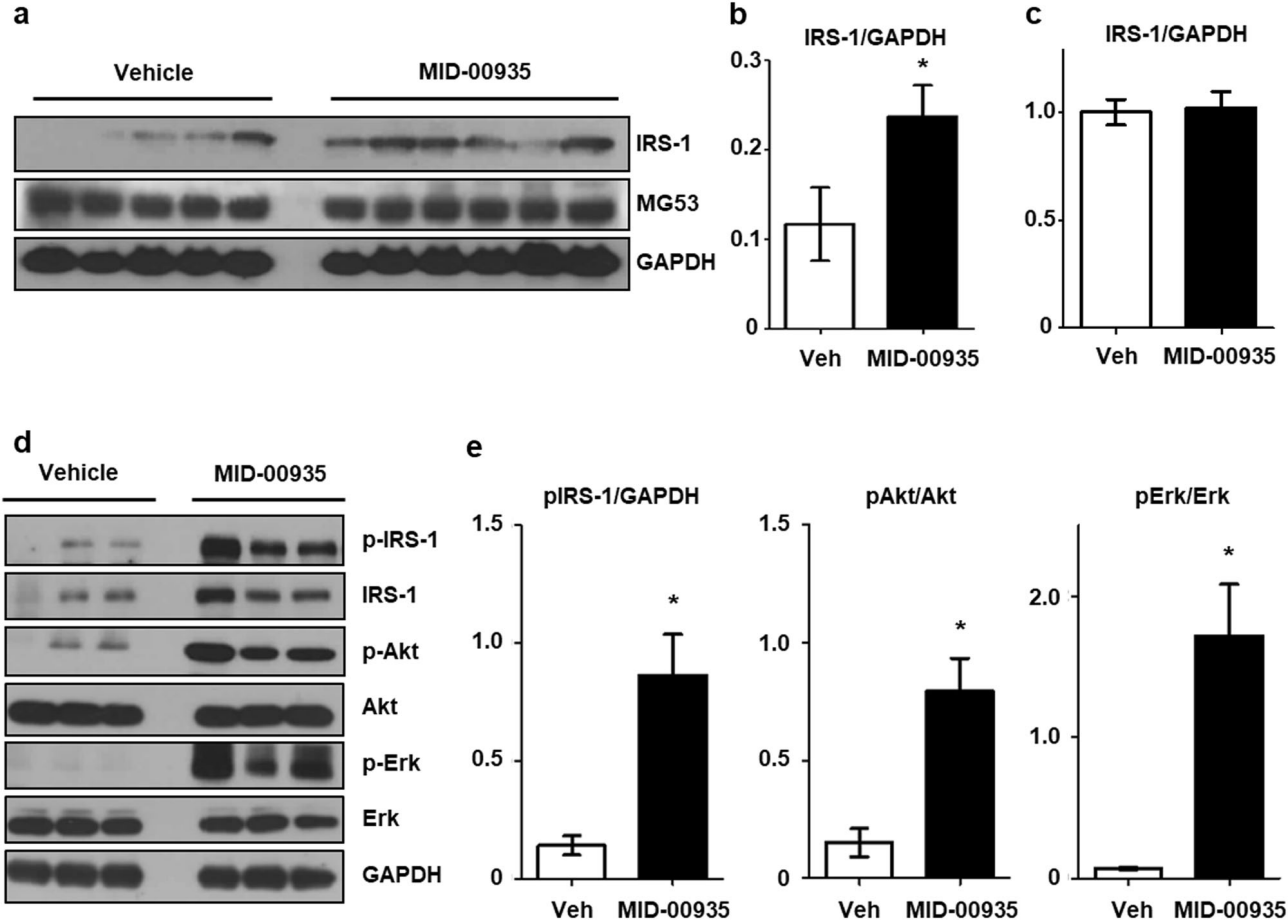
MID-00935 improves insulin signaling in the skeletal muscle of DIO mice. Four-week-old male mice were fed a high-fat diet for 10 weeks and orally treated with vehicle or MID-00935 (100 mpk, twice a day) for 2 weeks. Soleus muscle was isolated from vehicle- and MID-00935-treated DIO mice. The protein levels of IRS-1 and MG53 were determined by immunoblotting (**a**). Increased IRS-1 levels were statistically assessed (**b**). The mRNA level of IRS-1 was determined by qPCR (**c**) (*n* = 5 for the vehicle-treated group, and *n* = 6 for the MID-00935-treated group). Vehicle- or MID-00935-treated DIO mice were overnight-fasted, and soleus muscle was isolated at 10 min after an intraperitoneal insulin injection (10 U/kg of body weight). The expression levels of IRS-1, p-IRS-1, Akt, p-Akt, Erk, and p-Erk were determined by immunoblotting (**d**). p-IRS-1, p-Akt and p-Erk levels in the soleus muscle were statistically assessed (*n* = 3 for each group) (**e**). Data are presented as the means ± SEM; *p < 0.05 compared to the vehicle-treated group

We measured body weights and fasting glucose levels in DIO mice fed a high-fat diet for 8 weeks after MID-00935 administration (100 mpk, twice a day) for one week. As shown in Fig. 4a, b, MID-00935 treatment reduced body weights from 36 to 32 g and fasting blood glucose levels from 142 to 118 mg/dL. MID-00935 treatment significantly reduced serum insulin and leptin levels (Fig. 4c, d). MID-00935 treatment also improved glucose disposal in glucose and insulin tolerance tests in DIO mice (Fig. 4e, f). Next, we measured the expression levels of IRS-1 in the skeletal muscle of MID-00935-treated DIO mice. The protein levels of IRS-1 were doubled by MID-00935 treatment (Fig. 5a, b). However, the mRNA levels of IRS-1 were unchanged (Fig. 5c). Insulin-induced IRS-1, Akt, and Erk phosphorylation was determined by immunoblotting in the skeletal muscle of MID-00935-treated DIO mice. After insulin stimulation, p-IRS-1, p-Akt, and p-Erk levels were higher in MID-00935-treated DIO mice than in vehicle-treated DIO mice (Fig. 5d, e). These data suggest that MID-00935 disrupts with the MG53-IRS-1 interaction in vivo and may be used as a therapeutic agent for treating insulin resistance.

### MID-00935 increases energy expenditure

**Fig. 6:**
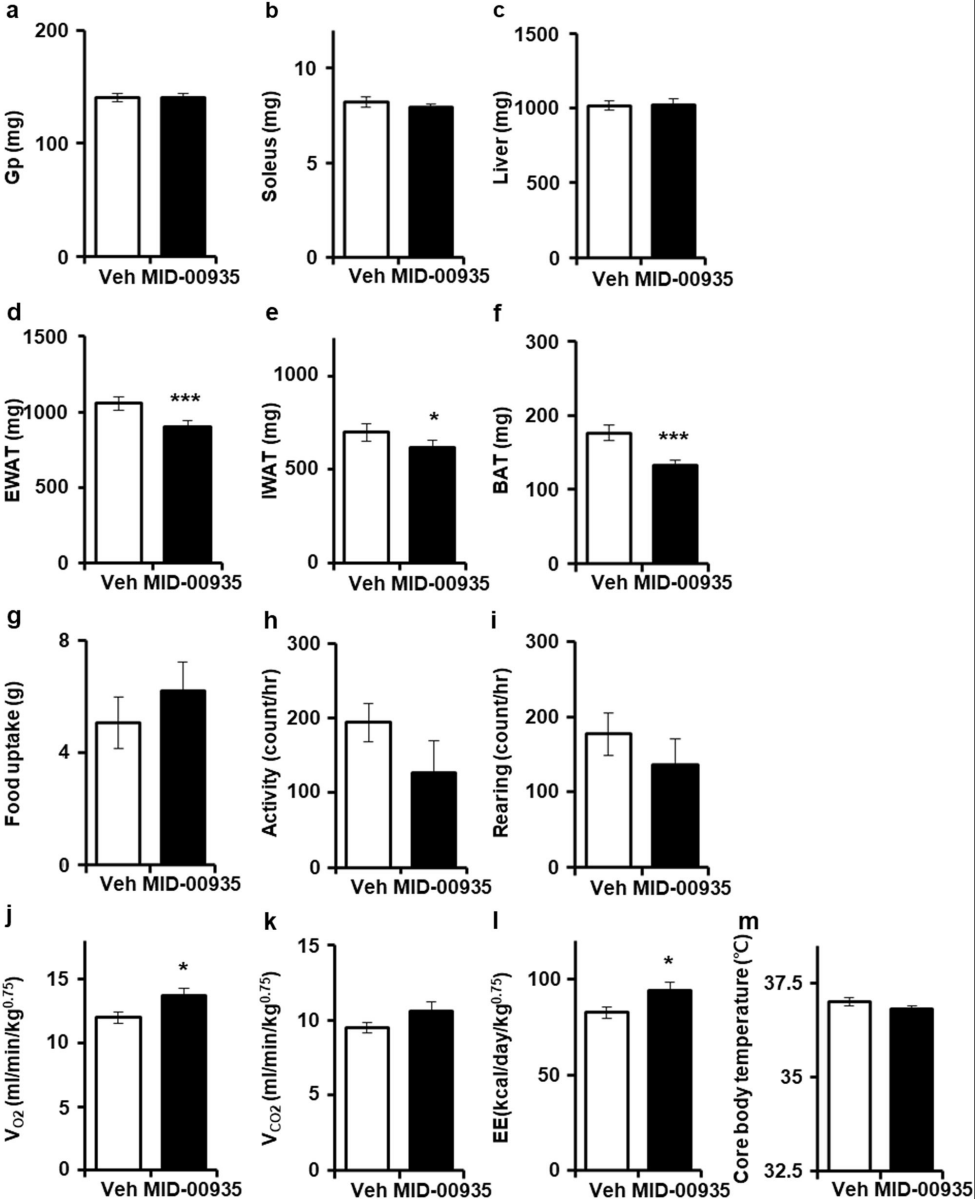
MID-00935 reduces fat mass and increases energy expenditure. Four-week-old male mice were fed a high-fat diet for 8 weeks and orally treated with vehicle or MID-00935 (100 mpk, twice a day) for one week. After an overnight fast, gastrocnemius plus plantaris (Gp), soleus muscle, and liver weights were measured (**a**–**c**). Weights of Epididymal (EWAT), inguinal (IWAT), and brown adipose tissue (BAT) were measured (*n* = 12 for each group) (**d**–**f**). Four-week-old male mice were fed a high-fat diet for 10 weeks and orally treated with vehicle or MID-00935 (100 mpk, twice a day) for 2 weeks. After treating DIO mice with vehicle or MID-00935 for 1 week, food uptake, locomotor activity, *V*_O2_, *V*_CO2_, and energy expenditure were measured for 24 h (*n* = 10 for each group) (**g**–**l**). Core body temperatures were measured by a thermocouple thermometer with a rectal probe (**m**). Data are presented as the means ± SEM; *p < 0.05 and ***p < 0.001, compared to the vehicle-treated group

Body weight reduction was significant after the oral administration of MID-00935 for 1 week (Fig. 4a). To know whether body weight reduction is an adverse effect or therapeutic effect of MID-00935, we measured muscle, liver, and fat mass. MID-00935 treatment did not change the skeletal muscle (gastrocnemius plus plantaris muscle and soleus muscle) or liver mass (Fig. 6a–c) but decreased the mass of epididymal and inguinal white adipose tissue and brown adipose tissue (Fig. 6d–f). These data showed that the body weight reduction effect of MID-00935 treatment results from fat mass reduction. Next, we determined food uptake, locomotor activity, core body temperature, and energy expenditure. MID-00935 treatment did not change food uptake, locomotor activity or core body temperature but increased oxygen consumption and energy expenditure (Fig. 6g–m). These results may indicate that MID-00935 treatment improves insulin sensitivity and glucose utilization in the skeletal muscle by increasing IRS-1 levels and whole body energy expenditure and by decreasing fat mass.

## Discussion

Skeletal muscle is the largest organ in the body; it accounts for ~40% of body weight and is responsible for 80% of insulin-induced glucose disposal from the blood^3, 4^. For the treatment of type 2 diabetes, exercise-mediated glycemic control via skeletal muscle is one of the major clinical guidelines in addition to dietary intervention and pharmacological glucose-lowering therapy^24, 25^. Exercise is as effective as metformin for treating T2DM^26^. However, there are no clinical drugs that directly target skeletal muscle to control blood glucose levels. For example, the first-line drug metformin increases AMPK activity and mitochondrial activity in skeletal muscle, but the primary glucose-lowering mechanism of metformin suppresses hepatic gluconeogenesis and glycogenolysis^27–29^. Because the peroxisome proliferator-activated receptor gamma (PPARγ) agonists thiazolidinediones (TZDs) ameliorate insulin resistance in skeletal muscle-specific PPARγ knockout mice, TZDs mainly improve insulin sensitivity by increasing fatty acid uptake in adipose tissue^30, 31^.

IRS-1 is tyrosine-phosphorylated and activated by the insulin-induced insulin receptor (IR). Activated IRS-1 stimulates the PI3K-Akt pathway, which induces GLUT4 translocation to the plasma membrane in skeletal muscle, leading to glucose uptake. The PI3K-Akt pathway in skeletal muscle also activates hexokinase, 6-phosphofructokinase, pyruvate dehydrogenase, and glycogen synthase, leading to glycolysis and glycogenesis^32^. Under diabetic conditions, IRS-1 levels are downregulated in various tissues, such as fat, the liver, the heart, endothelium, and muscle^33–38^. Genetic disruption of IRS-1 causes insulin resistance and diabetes^39, 40^. Thus, increasing IRS-1 levels in skeletal muscle may be an effective approach for treating insulin resistance.

MID-00935 increased IRS-1 levels and improved insulin sensitivity by interfering with the interaction between MG53 and IRS-1 (Figs. 2 and 3). The administration of MID-00935 improved insulin signaling and glucose uptake, with an increased IRS-1 level in skeletal muscle; these actions ultimately improved whole-body insulin sensitivity according to ITTs and GTTs (Figs. 4 and 5). Thus, MID-00935 may be developed as an anti-diabetic drug. Interestingly, human MG53 is expressed in skeletal muscle but not in cardiac muscle, whereas rodent MG53 is expressed in both skeletal and cardiac muscle^41^. Thus, MID-00935 may not exhibit any side effects on the human heart but may be synergistic with other tissue-targeting drugs, including metformin and TZDs.

Since insulin secretagogues and high-dose insulin therapy increase cancer incidence^42^, MID-00935 may also increase cancer incidence because MID administration improves insulin- and IGF-elicited Akt activation. Skeletal muscle-derived tumors are rare, and most of these are rhabdomyosarcomas^43^. Because rhabdomyosarcoma is derived from satellite cells that do not express MG53^44^, MID-00935 may not have any effect on the incidence of rhabdomyosarcoma. Thus, the therapeutic approach for targeting MG53 in skeletal muscle may be a useful way to treat type 2 diabetes without side effects.
